# Decreased Sexual Desire among Middle-Aged and Old Women in China and Factors Influencing It: A Questionnaire-Based Study

**DOI:** 10.1155/2021/6649242

**Published:** 2021-05-25

**Authors:** Ye Zhu, Xin Yang, Xiangling Fan, Yange Sun, Cheng Tan, Yanjie Wang, Wei Zhu, Dandan Ren

**Affiliations:** ^1^Peking University People's Hospital, Beijing, China; ^2^Beijing Second Hospital, Beijing, China; ^3^Yuetan Community Health Service Center under Fu Xing Hospital of Capital Medical University, Beijing, China; ^4^Peking University Third hospital, Beijing, China; ^5^Mudanjiang Medical University, Helongjiang, China

## Abstract

**Objective:**

This survey was designed and conducted with an aim to present data on sexual desire and activity in Chinese women.

**Methods:**

Between October 2013 and December 2013, we surveyed 3000 women (aged 40–65 years) at Beijing No. 2 Hospital and the Yuetan Community Health Service Center using a questionnaire. The primary outcomes included determination of sexual desire in the past 4 weeks, reasons for stopping sexual activity, and postmenopausal syndrome. The secondary outcome was determination of factors for low sexual desire.

**Results:**

A total of 2400 women (mean age 54.33 ± 6.25 years; mean menopausal age 50.11 ± 3.31 years) returned the questionnaire, with 58% of women reporting lowered sexual desire and 39.3% reporting stoppage of sexual activity. Compared with the postmenopausal group, the incidence of anxiety, depressive, somatic, and vasomotor symptoms was higher in the perimenopausal group. Muscle and joint pain (45.8%) and vaginal pruritus (21.5%) were the most commonly reported menopausal and vulvovaginal symptoms, respectively. The odds of decrease in sexual desire were significantly higher with older age, menopause, presence of gynecological disease, menopausal depression symptoms, menopausal vasomotor symptoms, and vulvovaginal atrophy; only cesarean delivery (odds ratio = 0.887, *P*=0.018) was associated with lesser reduction in sexual desire compared with the aforementioned factors.

**Conclusion:**

This survey showed that a high proportion of Chinese middle-aged and old women have lowered sexual desire and activity. Lack of sexual desire is associated with multiple factors and affects the quality of life of women.

## 1. Introduction

The concept of sexual health is a recent one and is defined by the World Health Organization as the condition of being physically, mentally, and socially well in relation to sexuality [1]. Furthermore, it has not been considered a priority, probably owing to limited clinical discussions on the topic [2]. Female sexual dysfunction (FSD), a highly prevalent condition, affects up to 63% of premenopausal women globally and has a higher prevalence in postmenopausal women (up to 86.5%) [3]. Ma et al. reported that 37.6% of women in China had FSD [4].

Hypoactive sexual desire disorder (HSDD) is described as “persistent absence of sexual fantasy or lack of sexual desire; and causing marked stress or interpersonal difficulties” by the 2016 International Society for the Study of Women's Sexual Health consensus [5]. As it is the most common of the 4 disorders in FSD and affects approximately 10% of adult women worldwide [5], it should be carefully assessed and treated by healthcare professionals [6].

Several studies have determined the prevalence of low sexual desire or HSDD and its risk factors [7–11]. In the study by West et al., the incidence of low sexual desire was lower in premenopausal women than in women with natural or surgical menopause (26.7% vs. 52.4% and 39.7%). However, a lower proportion of women with natural menopause reported HSDD compared with premenopausal women [7]. In a pan-Brazilian study by Abdo et al., the HSDD prevalence was 9.5%, and cardiovascular disease, posttraumatic stress, older age, no sex education, and forced and type of intercourse were significant risk factors [8]. Low sexual desire and HSDD in women with surgical menopause was also confirmed by Graziottin et al. and Rosen et al. [10, 11]. Factors associated with decrease in sexual desire and activity are old age, menopause [12, 13], physical health, social environment, culture, relation with partner [4], and decline in estradiol and testosterone levels [3, 14], despite the established fact that sexual desire and activity extend beyond 60 years of age [15, 16]. Levine described sexual motivation derives from individual psychological, biological factors, interpersonal issues, and cultural elements. According to Levine, culture plays an important role in sexual desire by programing the sexual mind. Biological components include age, hormonal levels, health issues, and medications. Sexual dysfunctions also have a negative effect on sexual desire [17, 18]. According to Basson, sexual desire in women may be influenced by past sexual experiences, that is, sexual response phases are related circularly [19]. Objectification is also considered as a cause of HSDD. Sexual objectification may lead to depression, disordered eating, and sexual dysfunction in female [20]. In a study by Steer et al., self-objectification was found to have correlation with body shame and appearance anxiety that develop self-consciousness during sexual activity which in turn lead to decreased sexual functioning [21]. In a recent study by Waite et al., men were reported to be more likely sexually active and have more positive permissive attitudes toward sex compared to women [22].

Till recently, only gynecological factors were studied in the Chinese female population, with little data on contraceptive use, menopause [23], and sexual health (sexual desire and activity aspects) [24]. However, recent studies have determined sexual activity and desire in Chinese women [25, 26]. The study by Zhang et al. included a small sample size (120 women) and compared the sexual activity in different age groups using the Female Sexual Function Index (FSFI) score [25]. A cross-sectional survey by Zhou et al. is the only study with a large sample size and determined the sexual activity in Chinese middle-aged women and associated risk factors [26]. As limited evidence is available in this context, we determined the prevalence of Chinese middle-aged and old women with low sexual desire, factors affecting sexual desire in the included population, and reasons for discontinuing sexual activity using a cross-sectional survey.

## 2. Materials and Methods

### 2.1. Study Design and Participants

This questionnaire-based survey was conducted to determine the prevalence of decreased libido (sexual desire) and factors influencing it in middle-aged and old Chinese women between October 2013 and December 2013 at the Administrative division, Beijing No. 2 Hospital, and the Yuetan Community Health Service Center, Fu Xing Hospital of Capital Medical University. The survey intended to include approximately 3419 participants. Investigators from the Peking University People's Hospital and Mudanjiang Medical University provided support for methodology development and statistics.

Women, aged 40–65 years, who had voluntarily participated in cervical cancer and breast cancer screening tests in the institutions were administered with a hard copy of survey questionnaire. Women with malignant tumors (breast and cervical) and those with iatrogenic menopause were excluded from the survey. All the participants signed an informed consent form before taking the survey. The trained staff informed subjects of the exclusion criteria and inclusion criteria. The questionnaire was filled by women in the hospital classroom. The staff checked to ensure that all the questions in the questionnaire were filled in (to avoid overlooking of questions). If the participants had any queries about the items in the questionnaire, the professional staff answered them on-site.

**Figure 1:**
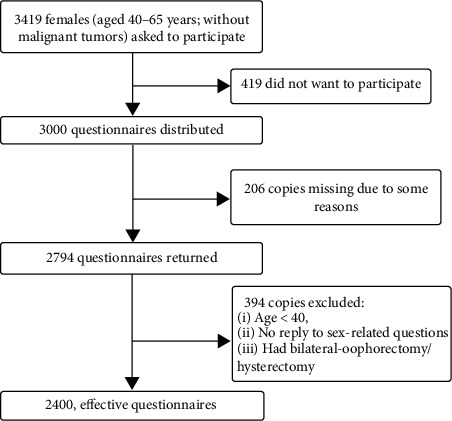
Survey participant disposition.

A copy of the survey questionnaire was administered to 3000 women; 419 women did not wish to participate in the survey. Overall, 206 questionnaires were not returned, and 394 questionnaires were excluded due to multiple reasons: age < 40 (*n* = 2), no reply to sex-related questions (*n* = 376), and had bilateral oophorectomy/hysterectomy (*n* = 29). Finally, 2400 effective and valid questionnaires were included for the study (effective response rate: 85.90%, Figure 1).

### 2.2. Survey Questionnaire

A self-administered questionnaire (to be filled by the patients) was used during the survey, which took approximately 20 minutes to complete. Before the survey, the investigators received training on forming a professional team that was familiar with the contents of the questionnaire and its interpretation. The questionnaire covered the following 4 aspects: (1) general and demographic information, (2) survey on sexual life, (3) Greene Climacteric Scale [27] for menopausal symptoms, and (4) vulvovaginal atrophy score [28, 29]. A bilingual expert with the help of medical experts (to ensure scientific accuracy) translated the questionnaires (FSFI, Greene climacteric score) used in the study to Chinese. To verify the understandability of the translated questionnaire, they were administered to small sample (25 participants). The responses obtained were translated to English by the experts for scientific presentation. To check linguistic equivalence, initial and final English versions were compared. For reliability, 30 participants were asked to complete translated questionnaire with an interval of 2 weeks, so that the participants do not remember the responses provided previously. Data regarding the comorbidities among the study participants were also collected. After completion of the survey, all the responses were evaluated by the investigators to ensure the completeness of the responses. The questionnaire about general and demographic information included age, weight, height, menstrual status, and income; menopausal medicines included hormonal therapy, Chinese medicine, and health products; physical disease included hypertension, diabetes, and heart disease; gynecological diseases included uterine fibroids, ovarian cysts, and benign cervical lesions; and gynecological surgery included surgery on the uterus, ovaries, and cervix, while survey on sexual life is based on FSFI scores. Greene Climacteric Scale included questionnaire about psychological, somatic, vasomotor symptoms, and sexual function abnormity.

### 2.3. Outcomes

The primary outcome was determination of decrease in sexual desire during the past 4 weeks based on the FSFI [30]. Other primary end points in our study included (1) reasons for stopping sexual activity, (2) comparison of severity of menopausal syndrome between peri- and postmenopausal participants based on the Greene Climacteric Scale scores, and (3) vulvovaginal atrophy score. The secondary outcomes in our study included (1) comparative analysis of patients with and without decreased sexual desire and (2) determination of factors for decreased sexual desire. We evaluated outcomes based on the past 4 weeks only, as the Greene Climacteric Scale required participants to report their feelings within 4 weeks.

In our survey, 2 questions on sexual desire were cited from the FSFI, with each question being scored on a scale of 1–5 (total score range: 2–10). With the coefficient of sexual desire being 0.6, the lowest and highest scores were calculated (factor × 2 or 10) as 1.2 and 6.0, respectively, and in the study, defined decrease in sexual desire as a total score of ≤3.6 points.

The third section of the questionnaire consisted of 21 questions that are divided into four parts: (1) psychological symptoms (Q1–Q11), (2) somatic symptoms (Q12–Q18), (3) vasomotor symptoms (Q19 and Q20), and (4) sexual function abnormity (Q21) from the Greene Climacteric Scale [31]. A 4-point Likert scale (0, no symptoms; 1, mild symptoms; 2, moderate symptoms; and 3, severe symptoms) was used to score the severity of symptoms. We defined “perimenopause” as a persistent difference of ≥7 days in the length of consecutive cycles. Persistence was defined as recurrence within 10 cycles of the first variable-length cycle [32], and “menopause” was defined as diagnosis after >12 months of amenorrhea [33] and may not be measurable by menstrual patterns in women with a history of hysterectomy or menopausal hormone therapy use [34]. The vulvovaginal atrophy score was determined using a vaginal health score without the pH test and was self-evaluated. The score comprised 4 factors: vaginal pain, dyspareunia, vaginal pruritus, and vaginal local burning sensation.

### 2.4. Statistical Analysis

Statistical analysis was performed using SPSS version 19.0 (IBM SPSS Statistics for Windows, Version 19.0. Armonk, NY : IBM Corp.). Descriptive statistics were used to present all the data, except the factors influencing the decrease in sexual desire. Categorical data were presented as number and frequency, that is, *n* (%), whereas continuous data were presented as means ± standard deviation (SD). The participants were divided into 2 groups, those “with decreased sexual desire” and those “without decreased sexual desire.” Between the groups, univariate analysis for categorical data (demographics, medical history, and contraception history) and continuous data (Greene Climacteric score and vulvovaginal atrophy score) were compared using the chi-square test and rank-sum test, respectively. A *P* value of ≤ .05 was considered statistically significant. Moreover, factors showing *P* ≤ 0.01 in the chi-square test and rank-sum test were included in the logistic regression analysis to determine factors influencing decreased sexual desire.

## 3. Results

### 3.1. Population Characteristics and Distribution

The survey sample comprised 2400 female participants (mean age 54.33 ± 6.25 years, mean weight 57.83 ± 12.38 kg, mean height 159.21 ± 0.05 cm, and mean menopausal age 50.11 ± 3.31 years).  Table 1 presents the distribution of participants across different groups. The participants were divided into 3 age groups: 40–49 years (20.5%), 50–59 years (55.0%), and ≥60 years (24.4%). Of the 2400 participants, 29.3% had regular menstrual status, 9.9% had irregular menstrual status, and 60.8% had already entered menopause (Table 1). Among the participating women, 39.3% had stopped sex completely and 75.8% women had gynecological diseases (Table 1). Menopausal medicines were being taken by 77.9% of the participants (Table 1). More than 75% of the participants were found to have at least 1 gynecological disease at baseline (Table 1). Common comorbid conditions among the participants included hypertension (27.7%), arthritis (22.0%), and dyslipidemia (24.7%). Incidences of comorbidities among the participants are given in Table 2.

### 3.2. Reasons for Decrease in Sexual Desire and Cessation of Sexual Activity

**Figure 2:**
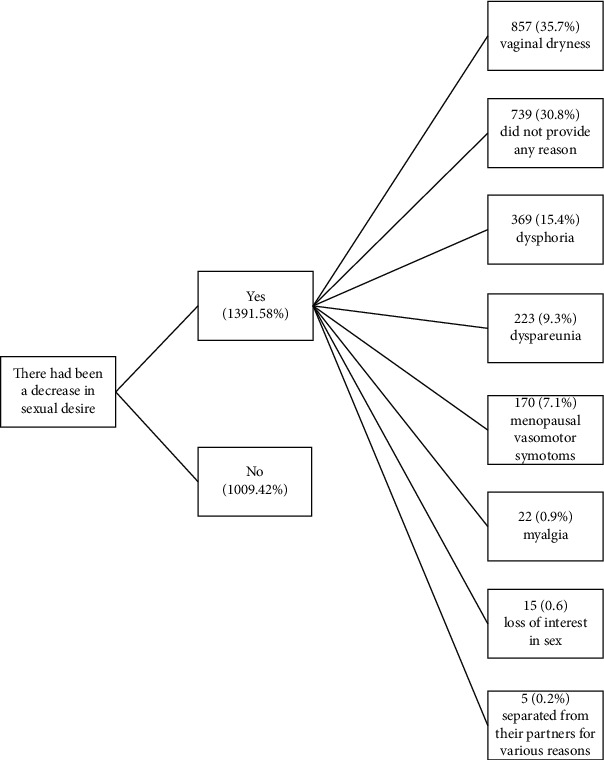
Proportion of patients with decrease in sexual desire, with reasons.

Overall, 58% of the respondents had decrease in sexual desire, with a mean FSFI score of 5.78 for sexual desire. The major reasons were vaginal dryness (35.7%), dysphoria (15.4%), and dyspareunia (9.3%). Approximately 31% of respondents did not provide any reason for the response (Figure 2).

In the 944 respondents (39.3%) who had completely stopped sexual activity, the major reasons were physical disease in any of the partner (17.1%), sexual dysfunction in any of the partner (15.8%), and unsafe environment (8.4%). No specific reason was provided by 49.2% women (Table 3). Furthermore, 207 respondents reported “disruption in life due to a sex-related problem” and reportedly sought solutions via books (43.48%) or the Internet (12.56%) and from doctors (19.32%), spouse (12.01%), friends (6.28%), or family members (8.21%).

### 3.3. Menopausal and Vulvovaginal Symptom Frequency

The mean scores for psychological, somatic, vasomotor, and sexual function abnormalities were 0.34, 0.36, 0.33, and 0.38, respectively. The mean overall score was 0.35. Menopausal symptom evaluation using the Greene Climacteric Scale showed that compared with postmenopausal participants, a greater proportion of perimenopausal participants reported a higher anxiety score (69.33% vs. 64.95%) and depressive score (66.81% vs. 59.05%). Somatic (71.85%) and vasomotor (43.28%) scores were also higher in the perimenopausal group than in the postmenopausal group (Table 4). No menopausal symptoms were reported by 485 (20.2%) participants. In terms of individual symptoms, the most frequently reported symptoms were “muscle and joint pain” (45.8%), “feeling tired or lacking in energy” (44.7%), and “difficulty sleeping” (40%). Among the vulvovaginal symptoms, vaginal pruritus (21.5%) was the most common symptom, followed by dyspareunia (16.3%), vaginal pain (10.1%), and vaginal local burning sensation (8.9%). Of the respondents, 1607 (67.0%) never had any vulvovaginal atrophy symptoms and 793 (33%) had at least 1 vulvovaginal symptom.

### 3.4. Factors Influencing Decrease in Sexual Desire

From univariate analysis (Table 5), factors such as age (*P* < 0.001), menstrual status (*P* < 0.001), body mass index (BMI; *P* < 0.001), presence of physical disease (*P* < 0.001), menopausal medicine usage (*P* < 0.001), presence of gynecological disease (*P*=0.01), and Greene Climacteric Scale and vulvovaginal atrophy scores (*P* < 0.001) significantly affected sexual desire.

Multivariate analysis showed that the odds of decrease in sexual desire were significantly increased with older age (odds ratio, OR = 1.328), menopause (OR = 1.20), presence of a gynecological disease (OR = 1.231), menopausal depression symptoms (OR = 1.738), menopausal vasomotor symptoms (OR = 1.328), and vulvovaginal atrophy (*P* < 0.05 for all; Table 6). However, cesarean delivery (OR = 0.887, *P*=0.018) lowered the odds of suppression of sexual desire.

## 4. Discussion

Sexual satisfaction has become an important indicator for measuring the quality of life (QoL) and a crucial aspect of studies on human sexual life in recent years [13, 35]. However, discussing sexual health, especially FSD or sexual activity, is still a taboo in Asian countries [15, 36]. As China is also a conservative country where research on sexual health, reproductive health, contraceptive use, and menopause-related problems has been limited [23], FSD or sexual health has been inadequately studied [25, 26]. Other studies in Hong Kong and China have also reported the prevalence and risk factors of FSD in young and middle-aged women [37, 38]. To the best of our knowledge, this study is among the very few studies that evaluated sexual desire and activity in middle-aged and old Chinese women and compared different sexual activity groups in terms of their demographic characteristics.

Based on the survey results, low sexual desire was observed in middle-aged and old Chinese women (58%). However, the prevalence of sexual desire and activity was lower than that reported in a previous study on Chinese women of a similar age group (72.74%) [26]. Moreover, Zhou et al. reported that sexual desire and activity decreased the most among postmenopausal women compared with peri- or premenopausal women [26]. Our study findings were also in agreement with the previous study results, in which the highest proportion of women aged ≥60 years (27.1%) and menopausal women (66.6%) showed low sexual desire and activity. The association between older age and lowered sexual desire is also confirmed from the findings of Zhang et al., where a greater proportion of middle-aged women lacked sexual interest and found it unpleasurable [37]. In contrary, Rosen et al. reported similar low sexual desire in premenopausal and postmenopausal women but lower sexual function index in postmenopausal women compared to postmenopausal women. As the study included women with diagnosed HSDD, the study might have missed data of women that does not seek help for their disorder [11].

A study on Iranian women with menopausal symptoms reported sexual dysfunction in 53%, evaluated using FSFI questionnaire [39]. Zeleke et al. reported low sexual desire in 88.0% of older women [40]. Decreased sexual function in postmenopausal women was also confirmed in a recent study by Pérez-Herrezuelo et al. [41]. Overall, from the literature, it is clear that an association exits between older age and lowered sexual desire.

In conservative societies, the proportion of women without sexual activity is ≥ 60% [15, 39, 42], and a high proportion of women discontinues sexual activity after menopause [42]. This shows that in addition to age or menopause, ethnic background may also be an important contributing factor for low sexual desire. In contrast, sexual desire has been extensively studied in the Western population; however, the results were similar, that is, the proportion of women with HSDD or low sexual desire increased with age and menopause [8–11, 40, 43]. In addition, FSFI scores, a well-accepted scale [44] for studying sexual desire in postmenopausal women or women aged ≥56 years, were significantly lower than those for premenopausal and younger women [25, 39, 43], thus corroborating our results. A recent study by Pérez-Herrezuelo validates the Spanish version of FSFI and also reported decreased sexual function among postmenopausal women [45].

Among the reasons cited for low sexual desire in our study by the women participants, vaginal dryness (35.7%) was the most common. The factors reported in our study for low sexual desire and activity, such as vaginal dryness and dyspareunia, loss of sexual interest due to painful experience during the intercourse, and absence of a partner, were in line with previous reports [3]. In our study, 15.4% of women reported postcoital dysphoria (PCD), whereas in another study, the presence of PCD symptoms showed an inverse relation with sexual activity [46]. Fibromyalgia, considered as another important factor for lower sexual desire [47], was reported in 0.9% of our study population. Alternatively, hyperfunction of the autonomic nervous system may also be associated with decreased sexual desire [48]. Vulvovaginal symptoms was also found to lower sexual activity (dyspareunia reported in 9.3% of women with low sexual activity), in accordance with previous studies [49–51]. Thus, the reasons for low sexual desire are extensive; however, the topic remains a taboo for discussion as women may be uncomfortable in sharing details of their personal or sexual life [3] with clinicians or even in a survey. In our survey also, 30.8% of women did not disclose the reason for decreased sexual activity, which was possibly due to hesitation in sharing personal details.

Of the women who participated in the current study, 39.5% had completely stopped engaging in sexual activity, which was lower than that reported in studies by Addis et al. [52] and Lindau et al. [53]. In the study by Addis et al., 71% of the participating women were sexually active, of which 37% had engaged in sexual activity in the last month; daily sexual activity was reported in 1% of women [52]. However, Lindau et al. reported that 33% (1026) of the women in their study were sexually active, which was similar to the findings of the present study [53]. Thus, the current study also confirms that sexual desire and activity are present in the elderly [15, 16] and essential for their QoL [35].

Influence on sexual function is multifactorial, with the factors varying for adolescents [54], adults [55], and middle-aged or elderly populations [15, 56, 57]. In our study, multivariate regression analysis showed older age, menopause, presence of gynecological disease, menopausal depression symptoms, menopausal vasomotor symptoms, and vulvovaginal atrophy as the factors associated with greater odds of lowering sexual desire and activity. Cesarean delivery was the only factor that did not hamper sexual desire in the participants of our study. Our results are in line with those of Zhou et al., in which age, menopause, and presence of diseases significantly lowered sexual function among middle-aged and old Chinese women [26]. From the studies, decline in estrogen and testosterone levels is a major factor affecting sexual desire. Other factors associated with increasing age such as physical, physiological, mental (or psychological), or interpersonal relation with the partner, lifestyle, and sexual activity during the early years are also responsible for altering the sexual function in women [3, 16, 58]. These findings were commonly observed across the studies where the presence of a disease (physical factor) [8, 50, 51, 59–61], partner-related factors [9, 38, 43], psychological factors [10], and lifestyle factors [54] were associated with a lower sexual function or lower proportion of women engaging in sexual activity.

Cesarean delivery was the only factor in our survey that showed positive association with sexual life. According to literature, planned cesarean delivery causes lower incidence of pelvic floor dysfunctions, thus not affecting sexual desire adversely [62]. Moreover, another study showed that cesarean delivery might not have long-term effects on sexual function [63]. However, ethnicity is another potential factor that may affect sexual activity; Caucasian and African-American women engage in more sexual activity than their Asian/Chinese counterparts [52, 64]. However, this variance in sexual activity may also be attributed to the cultural or societal outlook toward sex.

The survey had a few limitations. First, selection of only 2 communities and >2000 women as a representative sample size may not reflect the general sexual situation in Beijing or China. Second, most Chinese still consider sexual matters as private, and thus, many women did not respond to certain questions in the survey. Third, sex education is still limited and in nascent stages in China; some patients were confused when responding to sex-related questions. Providing sex education to individuals of all ages should be prioritized, and clinicians must also be trained to provide sex education effectively. Fourth, we did not include/evaluate noncoital sexual practices, the level of collaboration of their partners or aspects related to other facets of health, sexually associated distress, possible negative attitudes (their own or their partners) towards their aging process, and quality of life. Further studies are required to explore the topic of sexual desire and activity in middle-aged and old women.

### 4.1. Implications for Practice and/or Policy

The present study is one among the very few studies that evaluated sexual desire and activity in middle-aged and old Chinese women. The study provides insights in the prevalence of HSDD in postmenopausal women and importance of HSDD as a component of quality of life in postmenopausal women. As part of health awareness, women and men should be provided sex education to make them aware of its importance in maintaining QoL. Furthermore, clinicians should be able to adequately address sex-related queries and handle cases of sexual function, especially from middle-aged or older individuals, to promote a healthy attitude toward sex.

## 5. Conclusion

A large proportion of middle-aged and old women in China lack sexual desire and activity. Overall, 58% of the respondents had decrease in sexual desire, with a mean FSFI score of 5.78 for sexual desire. Lack of sexual desire is influenced by multiple factors such as vaginal dryness, dysphoria, and dyspareunia and because of a physical disease or sexual dysfunction in any of the partner; 39.3% of respondents in our study had completely stopped sexual activity.

## Figures and Tables

**Table 1:** Distribution of participants.

Variable	Value
Age (years), mean ± SD	54.33 ± 6.25
Age (years), *n* (%)	
** **40–49	493 (20.5)
** **50–59	1321 (55.0)
** **≥60	586 (24.4)
Menstrual status, *n* (%)	
** **Regular	704 (29.3)
** **Irregular	238 (9.9)
** **Menopause	1458 (60.8)
Monthly personal income (yuan), *n* (%)	
** **<1000	92 (3.8)
** **1000–1999	301 (12.5)
** **2000–2999	1135 (47.3)
** **3000–3999	502 (20.9)
** **4000–4999	166 (6.9)
** **>5000	204 (8.5)
Level of education, *n* (%)	
** **Junior middle school or lower	1062 (44.3)
** **Senior middle and secondary school	837 (34.9)
** **College or bachelor's degree	501 (20.9)
Body mass index, kg/m^2^, *n* (%)	
** **<18.5 (low bodyweight)	245 (10.2)
** **18.5–23.9 (normal bodyweight)	1737 (72.4)
** **≥24 (overweight or obesity)	418 (17.4)
Delivery mode, *n* (%)	
** **Vaginal labor	1739 (72.5)
** **Cesarean delivery	587 (24.4)
** **N/A	74 (3.1)
Frequency of sex, *n* (%)	
** **Stopped completely	944 (39.3)
** **<1 time per month	671 (27.9)
** **1-2 times per month	554 (23.1)
** **1-2 times per week	177 (7.4)
** **3-4 times per week	21 (0.9)
** **≥4 times per week	33 (1.4)
Physical exercise, *n* (%)	
** **Never	209 (8.7)
** **<1 time per month	175 (7.3)
** **1-2 times/month	214 (8.9)
** **1-2 times/week	374 (15.6)
** **3-4 times/week	310 (12.9)
** **4–6 times/week	156 (6.5)
** **Daily	962 (40.1)
Any physical disease^a^, *n* (%)	
** **≥1	1397 (58.2)
** **None	1,003 (41.8)
Gynecological diseases^a^, *n* (%)	
** **≥1	1819 (75.8)
** **No	581 (24.2)
Gynecological surgery, *n* (%)	
** **Yes	337 (14.0)
** **No	2063 (86.0)

**Table 2:** Incidence of physical diseases.

Diseases	*N* (%)
None	1003 (41.8)
High blood pressure	665 (27.7)
Heart disease	233 (9.7)
Hyperthyroidism	47 (2.0)
Hypothyroidism	67 (2.8)
Diabetes mellitus	257 (10.7)
Arthritis	527 (22.0)
Dyslipidemia	590 (24.7)
Cancer	47 (2.0)
Fibromyalgia	22 (0.9)
Other diseases	39 (1.6)

**Table 3:** Factors for discontinuing sex.

Reasons	(*n* = 944)	
	*n*	%
Physical disease (in any 1 partner)	161	17.1
Sexual dysfunction (in any 1 partner)	149	15.8
Living in unsafe environments	79	8.4
In bad marital relationships	52	5.5
Single	15	1.6
Having no sexual needs	12	1.3
Separated from partner for various reasons	6	0.6
Physical discomfort because of menopause	3	0.3
Work-related stress	1	0.1
Physical discomfort after surgery	2	0.2
No specific reason	464	49.2

**Table 4:** Greene score results.

Menstrual status score	Perimenopause (*n* = 238)		Postmenopause (*n* = 1458)	
	*n*	%	*n*	%
Anxiety score	165	69.33	947	64.95
Depression score	159	66.81	861	59.05
Somatic score	171	71.85	981	67.28
Vasomotor score	103	43.28	579	39.71

**Table 5:** Predictive factors for decreased sexual desire.

Variable		Decreased sexual desire (*n* = 1391)		Without decreased sexual desire (*n* = 1009)			Chi^2^ (*x*^2^/*z*)	*P* value
		*n*	%	*n*	%
Age, years						66.699		<0.001
** **40–49		207	14.9	286	28.3			
** **50–59		807	58.0	514	50.9			
** **≥60		377	27.1	209	20.7			
Menstrual status						55.802		<0.001
** **Regular		328	23.6	376	37.3			
** **Irregular		137	9.8	101	10.0			
** **Menopause		926	66.6	532	52.7			
Level of education						2.519		0.284
** **Junior middle school or lower		627	45.1	435	43.1			
** **Senior middle school and secondary school		489	35.2	348	34.5			
** **College or bachelor's degree		275	19.8	226	22.4			
Monthly personal income						9.843		0.080
** **<1000		41	2.9	51	5.1			
** **1000–1999		179	12.9	122	12.1			
** **2000–2999		663	47.7	472	46.8			
** **3000–3999		291	20.9	211	20.9			
** **4000–4999		105	7.5	61	6.0			
** **>5000		112	8.1	92	9.1			
BMI, kg/m^2^						45.198		<0.001
** **<18.5		91	6.7	150	15.2			
** **18.5–24		1031	75.4	675	68.2			
** **≥24		245	17.9	165	16.7			
Delivery mode						15.507		0.001
** **Vaginal labor		1046	75.2	689	68.3			
** **Cesarean delivery		307	22.1	280	27.8			
Physical exercise						11.249		0.082
** **Never		125	9.2	79	8.1			
** **<1 time per month		107	7.9	63	6.4			
** **1-2 times/month		116	8.6	91	9.3			
** **1-2 times/week		201	14.8	163	16.6			
** **3-4 times/week		178	13.1	124	12.7			
** **4–6 times/week		102	7.5	49	5.0			
** **Daily		525	38.8	411	41.9			
Contraceptive method						8.989		0.533
** **Oral contraceptive		44	3.5	23	2.5			
** **Condom		394	31.7	309	34.2			
** **Rhythm method		82	6.6	73	8.1			
** **IUD		474	38.2	313	34.7			
** **Tubal sterilization		47	3.8	40	4.4			
** **Other contraceptives		8	0.6	7	0.7			
** **No contraception		192	15.5	138	15.3			
Physical disease						23.910		<0.001
** **Yes		868	62.4	529	52.4			
** **No		523	37.6	480	47.6			
Use menopausal medicine						19.084		<0.001
** **Yes		351	25.2	179	17.7			
** **No		1040	74.8	830	82.3			
Suffering from gynecological disease						6.663		0.010
** **Yes		1081	77.7	738	73.1			
** **No		310	22.3	271	26.9			
Gynecological surgery						0.310		0.577
** **Yes		200	14.4	137	13.6			
** **No		1191	85.6	872	86.4			
Greene scale score								
** **Anxiety symptoms		—	—	—	—	51.077		<0.001
** **Depression symptoms		—	—	—	—	71.103		<0.001
** **Physical symptoms		—	—	—	—	26.578		<0.001
** **Vasomotor symptoms		—	—	—	—	47.081		<0.001
** **Vulvovaginal atrophy score		—	—	—	—	55.044		<0.001

**Table 6:** Multivariate analysis of predictive factors for lowering sexual desire in women.

Variable	OR	95% CI	*P* value
Age	1.328	1.125–1.569	<0.001
Menstrual status	1.200	1.075–1.340	<0.001
Cesarean delivery (yes vs. no)	0.887	0.652–0953	0.018
Gynecological disease (yes vs. no)	1.231	1.468–1.978	0.028
Menopausal depression symptoms (yes vs. no)	1.738	1.389–2.175	<0.001
Menopausal vasomotor symptoms (yes vs. no)	1.321	1.087–1.605	0.005
Vulvovaginal atrophy (yes vs. no)	1.585	1.308–1.920	<0.001

## Data Availability

The datasets used or analyzed during the current study are available from the corresponding author upon request.
